# Inpatient Dementia Mental Health Care in the UK: A National Mapping Survey of Ward Provision and Family Carer Involvement

**DOI:** 10.1002/gps.70266

**Published:** 2026-10-28

**Authors:** Nathanael Bean˒, Benjamin R. Underwood˒, Rosie Dunn, Juliet Gillam, Faye Watson, Emma Wolverson

**Affiliations:** ^1^ School of Clinical Medicine University of Cambridge Cambridge UK; ^2^ School of Medicine and Population Health University of Sheffield Sheffield UK; ^3^ Department of Psychiatry University of Cambridge Cambridge UK; ^4^ Cambridgeshire and Peterborough NHS Foundation Trust Cambridge UK; ^5^ Geller Institute of Ageing and Memory University of West London London UK

**Keywords:** caregiver support, dementia, family carer, legislation, medicine, mental health, mental health care, nursing

## Abstract

**Objectives:**

Inpatient mental health wards provide specialist care for people with dementia experiencing acute behavioural disturbance. The scale and configuration of this provision, and how these services involve family carers, have not previously been described at a national level. This study aimed to map ward provision across the UK and characterise existing policies and practices related to carer involvement.

**Methods:**

A national cross‐sectional online mapping survey was conducted across mental health wards providing care for people with dementia in the UK. Wards were identified through a structured multi‐source search strategy. The survey captured ward characteristics, patient profile, staffing and approaches to carer involvement. Quantitative data were analysed descriptively; Fisher's exact test was used to examine associations between categorical variables.

**Results:**

209 wards comprising over 3100 beds across 80 provider organisations were identified. 63 wards (30.1%) participated, reporting 801 patients. Most patients were detained under mental health or capacity legislation (87.8%) and few were expected to return home (14.8%). While all wards reported involving carers in clinical decision‐making, 44.4% indicated carers did not routinely attend MDT meetings and 30.2% did not routinely share care plans. Staff training in carer engagement was a consistent marker of greater carer involvement across multiple measures.

**Conclusions:**

This survey provides the first national picture of inpatient dementia mental health care in the UK, revealing a service under considerable pressure and a consistent gap between the basic infrastructure for carer involvement and its meaningful integration into clinical practice. Staff training in carer engagement was strongly associated with greater carer involvement, pointing to an actionable route to improvement.

## Key Points

- This survey provides the first national mapping of inpatient dementia mental health ward provision in the UK, identifying 209 wards and over 3100 beds across 80 provider organisations.
- While basic provisions for carer involvement are largely in place, meaningful integration of carers into clinical processes, including MDT meetings and care planning, remains inconsistent across wards.
- Most patients are detained under mental health or capacity legislation and are unlikely to return home following admission, yet support for carers during this transition remains inconsistent.
- Staff training in carer engagement was strongly associated with greater carer involvement and represents an important focus for service improvement.

## 1 Introduction

People with dementia may experience changes in their mood and behaviour, including anxiety, depression, sleep disturbance, hallucinations and disinhibition [1, 2, 3]. These experiences are often described using the term behavioural and psychological symptoms of dementia (BPSD); however, this terminology is contested, with ongoing debate about the appropriateness of language used to describe experiences that may reflect distress and unmet need [4, 5, 6]. For some people with dementia, distress escalates to periods of acute behavioural disturbance, including aggression and persistent refusal of care, necessitating inpatient psychiatric admission [7]. Within a proposed seven‐tiered model, roughly one in ten people with dementia experience very severe behavioural disturbance and approximately one in one hundred display behaviour classified as extreme [8], though point prevalence estimates vary widely across the literature [9, 10, 11, 12].

In the UK, older adult mental health wards provide specialist care for people with dementia whose acute presentations cannot be safely managed in the community. Admissions frequently occur involuntarily under mental health and capacity legislation, principally the Mental Health Act 1983 (as amended 2007) and the Mental Capacity Act 2005 in England and Wales, with equivalent legislation in the other home nations [13, 14, 15, 16]. These stays are often prolonged, averaging around 100 days [17] and typically involving individuals in the later stages of dementia with concurrent frailty and multimorbidity [18]. In this context, family carers, defined as those providing informal unpaid support to a person with dementia, play a central role in safe and effective care.

Carers hold detailed knowledge of the person's life history, values, routines, communication patterns, distress triggers and de‐escalation strategies; information rarely captured in clinical records and critical where verbal communication is impaired [19]. Carer involvement is associated with improved person‐centred outcomes [20, 21], and national guidance recommends that family members and carers are actively involved in care planning and decision‐making and that staff training incorporates an understanding of carers' needs and experiences [22]. Concerns about the exclusion of carers from inpatient settings have prompted initiatives to formalise their role within hospital care, including John's Campaign, which advocates open visiting rights for family carers of people with dementia [23].

BPSD are associated with caregiver distress, and admission often follows a prolonged and challenging period of escalation at home or in a care setting [24, 25, 26]. For many families, admission marks a permanent transition, with hospitalisation frequently resulting in discharge to a long‐term care placement [27]. Carers navigating this shift face simultaneous demands, including managing their own distress, frequently shaped by experiences of grief, loss, and trauma; structured carer support following inpatient admission has been shown to improve carers' wellbeing [27].

Despite this, evidence indicates that carers are not consistently involved or supported during inpatient dementia admissions. Carers describe feeling excluded following admission, with abrupt reductions in communication, limited opportunity to contribute to care planning, insufficient emotional and practical support, and confusion about mental health legislation [26, 28]. These experiences reflect broader systemic shortcomings: the Care Quality Commission's dementia strategy review emphasises that good dementia care requires carers to be listened to and included, yet nearly half of patients in mental health wards reported that no support had been offered to their family or carers during crisis care [29, 30]. That such gaps persist despite clear policy intent underlines the need to examine how inpatient services currently involve and support carers in practice.

The national landscape of inpatient dementia mental health provision, including how services involve and support family carers, remains poorly understood. Basic information such as the number of wards across both the private sector and the National Health Service (NHS) in the UK, or the number of people they care for, remains unavailable. The most substantial UK dataset published to date includes data from just four wards, highlighting the limited evidence base in this area [17].

The National Institute for Health and Care Research (NIHR) funded study Family Interventions in Dementia Mental Health Environments (FIND ME, ref NIHR161439) addresses this gap through a mixed‐methods approach. The first phase, reported here, is a national mapping survey of dementia mental health wards across the UK. It maps the national landscape of ward numbers, bed capacity and staffing, alongside patient characteristics including admission routes, legal status and length of stay. It also examines how wards structure carer involvement, covering visiting arrangements, communication practices, and the support and training offered to family carers. By establishing a baseline picture, the survey aims to identify both examples of good practice and areas where change is needed.

## 2 Materials and Methods

We conducted a national cross‐sectional online mapping survey of mental health wards providing care for people with dementia in the UK. In the UK, inpatient mental health care for people with dementia is typically provided across two ward models: specialist dementia wards, and mixed older people's mental health wards that admit people with dementia alongside patients with functional psychiatric conditions (e.g., depression, psychosis, bipolar disorder), both of which were eligible for inclusion.

No single national directory of such wards exists, so wards were identified through a structured, multi‐source search strategy, aiming to maximise coverage. This involved a systematic review of NHS Trust and Health Board websites, regulatory inspection reports, consultation with the national Inpatient Dementia Community of Practice, liaison with the Faculty of Old Age Psychiatry, the Faculty of the Psychology of Older People, and the Mental Health Nurse Academics Network, and identification of relevant independent‐sector providers through the study advisory group.

### 2.1 Survey Instrument

The survey was co‐developed by the research team in collaboration with the study's patient and public involvement advisory group, comprising eight people with lived experience of dementia mental health wards. It comprised mainly fixed‐response items, with a small number of free‐text questions, and was designed to be completed in approximately 15 minutes to minimise burden on respondents working in busy clinical environments. The survey captured information on ward characteristics including: number of beds, geographical location and type of service, patient profile and routes of admission, staffing composition, and current approaches to working with and supporting carers, including their involvement in care planning and decision‐making, and the support offered to families. The survey was piloted by clinical and academic experts and was subsequently refined to include further questions on carer support and inclusion, and relevant mental health legislation applicable to all four nations in the UK.

Participants were informed at the outset that the survey was designed to capture a snapshot of their ward on the day of completion, with questions on current patients and staffing referring specifically to that day. The survey was completed by the ward manager or an appropriate senior clinician with oversight of ward practice; ward name was collected in the survey to identify responses from the same ward, and where duplicate responses were received, these were identified and removed manually prior to analysis.

### 2.2 Survey Distribution

All correspondence was sent from an institutional study email account and included a participant information sheet outlining the purpose of the study, the voluntary nature of participation, and the arrangements for data handling. The survey was open from 15 May 2025 to 23 January 2026 and was distributed to 53 NHS Trusts in England, 14 NHS Boards in Scotland, seven Health Boards in Wales, six Health and Social Care Trusts in Northern Ireland, and seven private providers.

### 2.3 Data Handling and Analysis

Quantitative data were analysed descriptively to summarise patterns in service configuration, staffing, and carer‐related practices across wards. Free‐text responses were analysed using qualitative content analysis to identify common approaches and areas of variation [31]. This approach was selected due to the relatively brief and structured nature of the free‐text data. Fisher's exact test was used to examine associations between categorical variables, due to small sample sizes and expected cell frequencies below five in several contingency tables. Odds ratios with 95% confidence intervals were calculated as measures of effect size.

### 2.4 Ethics

Ethical approval for the study was granted by the London Camberwell St Giles Research Ethics Committee and the Health Research Authority (REC Ref: 25/LO/0040). Completion of the survey was taken as informed consent. No personal‐level data were collected, and all data were stored and managed in accordance with UK data protection regulations.

## 3 Results

### 3.1 National Landscape

Our multi‐source search identified 209 wards comprising over 3100 beds across 80 provider organisations: 143 wards in England, 29 in Scotland, 19 in Wales, seven in Northern Ireland, and 11 across six independent‐sector providers (mean ward size 15.8 beds). This represents a substantial and previously undescribed national resource. Full characteristics of identified wards, participating wards, and findings related to carer involvement are summarised in Table 1.

**TABLE 1:** Ward, patient and carer‐related characteristics of participating wards.

Nation/provider	Wards (*n*)	Beds (*n*)	Providers (*n*)
National landscape: Inpatient dementia mental health wards identified in the UK			
England (NHS)	143	2315	52
Scotland (NHS)	29	446	11
Wales (NHS)	19	174	6
Northern Ireland (HSC)	7	42	5
Independent sector	11	142	6
Total	209	3119	80

Characteristic	*n* (%)
Ward characteristics and staffing (*n* = 63 wards; 958 beds)	
Ward type	
NHS wards	59 (93.7%)
Mixed‐sex ward	50 (79.4%)
Dementia‐specific ward	28 (44.4%)
Mixed older people's mental health ward	35 (55.6%)
Age policy	
Strict ≥ 65 age threshold	3 (4.8%)
≥ 65 as guideline (younger patients accepted under criteria)	23 (36.5%)
No fixed lower age limit	37 (58.7%)
Staffing	
Fully staffed on survey day	56 (88.9%)
Bank staff used	37 (58.7%)
At least one patient on one‐to‐one observation	44 (69.8%)
Patient characteristics (*n* = 801 patients)	
Demographics	
Male	476/798 (59.6%)
Female	322/798 (40.4%)
At least one patient aged under 65 on ward	41/63 wards (65.1%)
At least one patient aged over 90 on ward	27/63 wards (42.9%)
Ethnicity	
White (British, Irish, or other)	750/801 (93.6%)
Asian or Asian British	22/801 (2.7%)
Black, African, Caribbean or Black British	13/801 (1.6%)
Other ethnic group	11/801 (1.4%)
Mixed or multiple ethnic groups	5/801 (0.6%)
Admission sources	
Admitted from own home	270/801 (33.7%)
Admitted from acute general hospital	191/801 (23.8%)
Admitted from residential care home	144/801 (18.0%)
Admitted from nursing home	143/801 (17.9%)
Discharge destinations (*n* = 831 reported destinations)	
Expected discharge to nursing home	413/831 (49.7%)
Expected discharge to residential care home	157/831 (18.9%)
Expected to return to own home	123/831 (14.8%)
Discharge destination uncertain	112/831 (13.5%)
Legal status and observation	
Informal (voluntary) admission	98/801 (12.2%)
Detained under Section 3, Mental Health Act 1983	349/801 (43.6%)
Detained under Section 2, Mental Health Act 1983	120/801 (15.0%)
On one‐to‐one or constant observation	113/801 (14.1%)
Wards with longest admission over 1 year	20/63 wards (31.7%)
Carer access and visiting (*n* = 63 wards)	
Carers permitted to visit	62 (98.4%)
Flexible/open visiting policy	41 (65.1%)
Dedicated visitors' room available	45 (71.4%)
John's Campaign formally adopted	22 (34.9%)
Carer involvement and communication (*n* = 63 wards)	
Formal involvement in care	
Carers involved in clinical decisions and discharge planning	63 (100%)
Carers routinely attend MDT meetings	35 (55.6%)
Care plans routinely shared with carers	44 (69.8%)
Confidentiality/information‐sharing protocols in place	62 (98.4%)
Carer involvement documented in clinical record	62 (98.4%)
Communication methods	
Telephone and face‐to‐face contact	63 (100%)
Email	48 (76.2%)
Letters	40 (63.5%)
Text messaging	6 (9.5%)
Structured carer support and staff training (*n* = 63 wards)	
Carer needs assessment conducted	46 (73.0%)
Signposting to external carer support services	61 (96.8%)
Carer support or peer group available on ward	35 (55.6%)
Staff training in carer engagement	28 (44.4%)

*Note:* HSC = Health and Social Care Trust (Northern Ireland). MDT = multidisciplinary team. Bed totals are for the 197 wards with published bed numbers. Sex was reported for 798 of 801 patients. A further 28 patients (3.5%) were admitted from other specialist providers and 25 (3.1%) from other or unknown sources. Discharge destination percentages use the 831 expected destinations reported by wards (including ‘uncertain’; a further 3.0% were to other locations and 0.1% to hospice); some wards reported more destinations than patients.

### 3.2 Ward Characteristics and Staffing

Of the 209 eligible wards, 63 (30.1%) participated (59 NHS, four independent), reporting 958 beds (mean 15.2, range 8–27). Most were mixed‐sex (79.4%), with 20.6% single‐sex. Just over half (55.6%) admitted both patients with dementia and older adults with functional psychiatric illness, while 44.4% were dementia‐specific. Only a minority of wards operated under a strict ≥ 65 age threshold (4.8%); over a third (36.5%) used this as a guideline while accepting younger patients under defined criteria such as younger‐onset dementia, frailty or bed pressures, and most (58.7%) had no fixed lower age limit.

Most wards were fully staffed on the survey day (88.9% at full establishment, 92.1% considering staffing adequate). Day shifts typically included two to three registered mental health nurses and four to eight healthcare support workers, with registered general nurses present on 34.9% of wards. Bank and agency staff (nursing and healthcare support workers) were used by 58.7% and 6.3% of wards respectively. All wards reported access to core multidisciplinary roles including medical staff, occupational therapy, pharmacy and psychiatry, generally via sessional or on‐call arrangements.

### 3.3 Patient Characteristics

The 63 wards reported 801 patients under their care: 59.6% male and 40.4% female (sex recorded for 798); no patients identified as transgender or other. The patient population was predominantly White (93.6%, identified as White British, White Irish or White Other), with 2.7% identifying as Asian or Asian British, 1.6% as Black or Black British, 0.6% as Mixed or Multiple ethnicity, and 1.4% as another ethnic group. Patient ages ranged from 44 to 97 years, with 65.1% of wards caring for a patient aged under 65 and 42.9% for a patient aged over 90. The longest current admissions ranged from eight weeks to 13 years (mean approximately 519 days); 31.7% of wards reported at least one patient admitted for over a year.

Admission sources included 33.7% of patients from their own homes, 23.8% from acute general hospitals, 17.9% from nursing homes, 18.0% from residential care homes, and 3.5% from other specialist providers, with 3.1% from other or unknown sources. Wards reported 831 discharge destinations; most were expected to transfer to long‐term care, with 49.7% to nursing homes and 18.9% to residential care homes. Only 14.8% were expected to return home. Patients on mixed wards were more likely to have some expected home discharges than those on dementia‐specific wards (78.8% vs. 50.0% of the 61 wards reporting destinations, OR = 0.27, 95% CI 0.09–0.82, *p* = 0.030). Destinations remained uncertain for 13.5%, with 3.0% expected to discharge to other locations and 0.1% to hospice care.

Only 12.2% of patients were admitted voluntarily; most were detained under mental health or capacity legislation, including 43.6% under Section 3 and 15.0% under Section 2 of the Mental Health Act 1983, with additional patients detained under equivalent frameworks in Scotland and Northern Ireland. Dementia‐specific wards were significantly more likely to have an entirely detained population (71.4% vs. 34.3%, OR = 4.79, 95% CI 1.63–14.06, *p* = 0.005). A total of 14.1% of patients were on one‐to‐one or constant observation, with 69.8% of wards having at least one such patient.

### 3.4 Carer Access and Visiting Arrangements

Family carers were almost universally permitted to visit (98.4%), typically through standard in‐person arrangements without physical barriers beyond usual ward routines such as mealtimes or medication rounds; the sole exception (1.6%) used a visiting pod, in which contact occurs via a room off the ward. Visiting hours were mostly flexible: 65.1% of wards described open or personalised visiting policies with minimal restrictions, while 34.9% had fixed visiting times. Almost all (98.4%) wards provided written information to carers on admission such as welcome packs or guides. Physical facilities varied; 71.4% had a dedicated visitors' room while 28.6% used day areas or patient rooms. John's Campaign was reported as implemented by 34.9% of wards, with 52.4% explicitly reporting non‐implementation; 6.3% followed its principles without formal adoption and 6.3% were unaware of it.

### 3.5 Carer Involvement and Communication

Carer participation in formal ward processes was variable. All wards reported involving carers in clinical decision‐making and discharge planning, yet 44.4% indicated carers did not routinely attend MDT meetings or did so only sometimes, and 30.2% did not routinely provide carers with copies of care plans. All wards communicated with carers by telephone and face‐to‐face contact; most also used email (76.2%), letters (63.5%), and text messaging (9.5%). Nearly all wards had confidentiality and information‐sharing protocols (98.4%), usually based on consent, capacity or best‐interest decisions, and 98.4% documented carer involvement in the clinical record.

Dementia‐specific wards were significantly more likely to have a dedicated visitor room (89.3% vs. 57.1%, OR = 6.25, 95% CI 1.58–24.65, *p* = 0.006), but no significant differences were found between ward types in routine MDT attendance by carers (64.3% vs. 48.6%, OR = 1.91, 95% CI 0.69–5.28, *p* = 0.308), sharing of care plans (67.9% vs. 71.4%, OR = 0.84, 95% CI 0.29–2.49, *p* = 0.788), carer needs assessment (82.1% vs. 65.7%, OR = 2.40, 95% CI 0.73–7.91, *p* = 0.166), or adoption of John's Campaign (32.1% vs. 37.1%, OR = 0.80, 95% CI 0.28–2.29, *p* = 0.792).

### 3.6 Structured Carer Support and Training

Support for carers' own needs was inconsistently provided. Most wards (73.0%) conducted carer needs assessments, typically through a formal assessment or MDT discussion, while 27.0% did not. Formal peer or group support was less consistently available: 55.6% ran carer support or peer groups including carers' cafés, psychology‐led groups and monthly forums, and 44.4% did not. Signposting to external support was near‐universal (96.8%), with wards referring carers to local carers' centres, Admiral Nurses (specialist dementia nurses), dementia advisers and psychological support services. Several wards additionally reported dedicated carer lead roles, carer drop‐in sessions, psychology‐led support pathways and implementation of Triangle of Care, a framework promoting carer partnership and staff training in carer engagement [32].

Staff training in carer engagement was strongly associated with formal carer inclusion. Wards reporting such training (44.4%) were significantly more likely to share care plans with carers (85.7% vs. 57.1%, OR = 4.50, 95% CI 1.29–15.75, *p* = 0.026) and to have a designated carer lead role (67.9% vs. 31.4%, OR = 4.61, 95% CI 1.58–13.39, *p* = 0.005), and showed a consistent trend towards greater carer involvement across other measures including MDT attendance (67.9% vs. 45.7%) and availability of peer support groups (67.9% vs. 45.7%), though these differences did not reach statistical significance.

## 4 Discussion

Our survey, though not exhaustive, identified nearly 1000 beds around the country providing specialist dementia and mental health provision, a number equivalent in size to a large teaching hospital. Across participating wards, the survey revealed high bed occupancy, prolonged lengths of stay, and heavy reliance on bank staff, all indicative of the extent of pressure on this service. The prevalence of one‐to‐one observations, reported in over two‐thirds of participating wards, warrants particular attention. Constant observation is costly, disruptive to the therapeutic environment, and for people with dementia can itself be distressing, representing an unfamiliar presence that may exacerbate agitation rather than reduce it [33]. Investment in higher baseline staffing and specialist dementia training, enabling staff to anticipate distress and intervene early rather than resort to reactive containment, could reduce the need for one‐to‐one observation, improving the experience of care while reducing costs.

While wards widely recognised the value of family carers and made basic provisions for them, there was substantial inconsistency in how fully carers were integrated into care. Open visiting arrangements, known to foster trusting relationships and improve communication between staff, patients, and carers [23], were widely adopted, yet only a minority of wards had formally adopted John's Campaign [34], suggesting that although flexible visiting was frequently permitted, it was not consistently embedded as a core expectation of care. This is notable given that Regulation 9A of the Health and Social Care Act 2008 establishes visiting as a fundamental standard [35], yet does not extend to patients detained under mental health legislation, who constitute the majority of patients on these wards. Voluntary adoption of open visiting principles through frameworks such as John's Campaign is therefore all the more important in this setting.

The deeper involvement of carers in clinical decision‐making was less consistent. Over two‐fifths of wards did not routinely include carers in multidisciplinary team meetings and over three in ten wards did not routinely provide them with copies of care plans, suggesting variation in how information‐sharing policies are applied in practice. While confidentiality protocols governing information sharing were almost universally in place, they were commonly described as being applied on a case‐by‐case basis, guided by consent and capacity. Such discretionary approaches may lead to more cautious or inconsistent sharing of information with carers. Indeed, concerns about confidentiality are a well‐documented obstacle to carer involvement [36].

Disparities were also evident in the extent to which wards supported carers' own needs. Almost all wards signposted carers to external support services and provided information packs, consistent with best‐practice guidance that services should actively direct carers to available resources [29], yet over a quarter did not conduct a formal needs assessment, despite recommendations that staff should identify and address these proactively [37].

Taken together, these findings point to a dissociation between routine, superficial carer engagement and meaningful integration into care planning and support structures, consistent with evidence that family caregivers in mental health settings frequently report feeling marginalised despite policy rhetoric [17, 36, 38]. The Triangle of Care framework, which recommends that all staff receive training in carer engagement and appreciate carers' contributions and needs [32], represents one such policy commitment not yet uniformly translated into practice, with fewer than half of wards reporting relevant staff training in carer engagement.

The admission and discharge patterns observed here underscore the significance of these gaps. More patients were admitted from home than ultimately returned there, consistent with evidence that hospitalisation frequently precipitates long‐term care placement for people with dementia [27]. Admission thus often marks a transition from community living to institutional care, during which carer input is essential for safe and timely discharge and carers' own support needs are simultaneously at their greatest. At this stage of dementia, the carer is often the only person who can convey the individual's life story and how this should shape their care; without this input at these points of transition, there is a risk to the person's wellbeing, dignity and personhood. The difficulty of securing appropriate step‐down placements for individuals with complex dementia may complicate this transition [39], with ward managers reporting that the longest current hospital stays average over a year, suggesting a lack of suitable discharge destinations within the community.

Many wards reported accepting patients outside a strict older‐age threshold; significant variation in presentation and care needs across different ages may result in difficulty ensuring staff are adequately skilled to meet the needs of every patient they encounter. Furthermore, over half of participating wards were not dementia‐specific, suggesting that inpatient dementia admissions are often managed outside dedicated dementia services, with general older adult mental health wards frequently meeting this demand [40]. Mixed wards must manage a broader range of clinical conditions, which may in turn shape ward routines and expectations around family engagement, though we found no statistically significant differences between ward types in carer involvement practices. Dementia‐specific wards were more likely to have a dedicated visitor room, suggesting they may be structurally better suited to supporting carer access; yet this did not translate into greater involvement in clinical processes such as MDT attendance, care planning or carer needs assessment, consistent with previous work finding minimal differences in care quality between ward models [41].

Several limitations of this survey should be acknowledged. The findings reflect the perspectives of ward managers rather than carers themselves, and self‐report bias may mean that practices are described more favourably than they are experienced in reality. The survey was cross‐sectional in design, providing a snapshot of current provision rather than capturing how practices have changed over time. While the multi‐source identification strategy aimed to maximise coverage, it is possible that some wards were not identified or chose not to participate, and the findings may therefore not be fully representative of all inpatient dementia provision across the UK.

These findings point to several clear priorities for future research and service development. Qualitative research exploring carers' own experiences during inpatient dementia admissions is needed to complement these findings and examine the extent to which ward‐level practices translate into meaningful inclusion in reality. The striking gap between home admissions and home discharges warrants further investigation, particularly around how carers are involved in placement decisions and supported through this transition. Finally, given that staff training emerged as a strong predictor of carer involvement, future work should examine which training models are most effective and how uptake can be increased at the ward level.

## 5 Conclusion

This survey maps a substantial and previously undescribed resource of 209 wards and over 3100 beds across 80 provider organisations and reveals that while the foundations for carer involvement are widely in place, meaningful integration into clinical processes remains inconsistent across care planning, needs assessment and staff training. Wards that had invested in staff training showed markedly greater carer involvement across multiple measures, pointing to a clear and practical route to improvement. For carers, inpatient admission frequently marks the beginning of a relative's permanent move to long‐term care, representing both a period of heightened need and a critical juncture at which services can, and should, engage carers as active partners. Achieving this requires not only policy commitment, which already exists, but consistent implementation at ward level, supported by training and a culture that recognises carers as essential to good care.

## Funding

This study was funded by the National Institute for Health and Care Research (NIHR), grant reference NIHR161439. The views expressed are those of the authors and not necessarily those of the NIHR or the Department of Health and Social Care.

## Ethics Statement

Ethical approval for this study was granted by the London Camberwell St Giles Research Ethics Committee and the Health Research Authority (REC Ref: 25/LO/0040). Completion of the survey was taken as informed consent.

## Consent

No personal or patient‐level data were collected in this study. Survey completion by ward managers or senior clinicians was taken as informed consent to participate.

## Conflicts of Interest

The authors declare no conflicts of interest.

## Data Availability

The data that support the findings of this study are available from the corresponding author upon reasonable request.
